# Enhancing maize growth and resilience to environmental stress with biochar, gibberellic acid and rhizobacteria

**DOI:** 10.3389/fpls.2024.1396594

**Published:** 2024-08-06

**Authors:** Tauseef Anwar, Huma Qureshi, Muhammad Saeed Akhtar, Ejaz Hussain Siddiqi, Hina Fatimah, Wajid Zaman, Bushra A. Alhammad, Mahmoud F. Seleiman

**Affiliations:** ^1^ Department of Botany, The Islamia University of Bahawalpur, Bahawalpur, Pakistan; ^2^ Department of Botany, University of Chakwal, Chakwal, Pakistan; ^3^ School of Chemical Engineering, Yeungnam University, Gyeongsan, Republic of Korea; ^4^ Department of Botany, University of Gujrat, Gujrat, Punjab, Pakistan; ^5^ Department of Biology, Allama Iqbal Open University, Islamabad, Pakistan; ^6^ Department of Life Sciences, Yeungnam University, Gyeongsan, Republic of Korea; ^7^ Biology Department, College of Science and Humanity Studies, Prince Sattam Bin Abdulaziz University, Al Kharj, Riyadh, Saudi Arabia; ^8^ Department of Plant Production, College of Food and Agriculture Sciences, King Saud University, Riyadh, Saudi Arabia; ^9^ Department of Crop Sciences, Faculty of Agriculture, Menoufia University, Shibin El-Kom, Egypt

**Keywords:** Phytoremediation, germination, oxidative stress, chlorophyll, soil amendment, resilience

## Abstract

**Background:**

*Zea mays* (maize) is a globally significant cereal crop with diverse applications in food, feed, and industrial products. However, maize cultivation is often challenged by environmental stressors such as heavy metal toxicity and drought stress (DS). Heavy metals like cadmium (Cd) and lead (Pb) can accumulate in soil through industrial activities and improper waste disposal, posing significant threats to plant growth and development. Drought stress further exacerbates these challenges by limiting water availability and affecting physiological processes in plants. This study explores the impact of Cd and Pb toxicity, as well as DS, on maize growth and development, and investigates the potential mitigating effects of various treatments, including gibberellic acid (GA3), biochar (BC), rhizobacteria (RB), and their combinations.

**Methods:**

The experiment involved maize plants subjected to different stress conditions: cadmium (Cd) at concentrations of 0, 6, and 12 ppm, lead (Pb) at 0 and 400 ppm, and drought stress (DS). Treatments included the application of 10 ppm GA3, 0.75% BC, a combined treatment of 10 ppm GA3 and 0.75% BC, rhizobacteria (RB), and a combined treatment of 0.5% BC and RB. The study measured germination rates, shoot and root lengths, and biochemical parameters such as shoot and root protein, phenolics, and chlorophyll contents under these conditions.

**Results:**

In the absence of Cd stress (0 Cd), the application of 10 ppm GA3 and 0.75% BC significantly enhanced germination rates by 72% and 76%, respectively, compared to the control, with the combined treatment exhibiting the highest enhancement of 86%. Under Cd stress (6 ppm Cd), GA3 and BC individually improved germination by 54% and 57%, respectively, with the combined treatment showing the largest increase of 63%. Drought stress influenced germination, with notable improvements observed with the application of 0.5% BC (50% increase) and RB (49% increase). Similar trends were observed in shoot and root lengths, where the combined treatment of GA3 and BC resulted in the most significant improvements. The treatments positively influenced shoot and root protein, phenolics, and chlorophyll contents, particularly under stress conditions.

**Conclusion:**

These findings highlight the potential of combined treatments, such as the application of GA3 and BC or BC with RB, in alleviating the adverse effects of heavy metals (Cd and Pb) and drought stress in maize cultivation. The combined treatments not only improved germination rates but also significantly enhanced shoot and root growth, as well as important biochemical parameters under stress conditions. This suggests that GA3 and BC, alone or in combination with RB, can play a crucial role in enhancing maize resilience to environmental stressors. The study highlights the importance of exploring sustainable agricultural practices to mitigate the impacts of heavy metal toxicity and drought stress. Future research should focus on long-term field trials to validate these findings and further investigate the mechanistic pathways involved in stress mitigation by these amendments, as well as their economic feasibility and environmental impact on a larger scale to ensure their practical applicability in real-world agricultural settings.

## 1 Introduction

*Zea mays* (maize), belonging to the Poaceae family, is cultivated worldwide and is one of the most important globally farmed grain crops (Abbas et al., 2017; Singh et al., 2017). By the end of 2020, maize yield per acre is expected to rise from the current level of 1.7 tons to 2.36 tons, addressing the increasing demand. Beyond its grain, maize oil, produced through the wet milling process, makes up approximately 45-50% of its total weight (Siyuan et al., 2018). This oil is used widely in cooking and salad dressings and is notable for its rich content of beneficial saturated fatty acids, which make up about 14% of its components. Additionally, when converted into hay and silage, maize is an essential component of livestock feed, particularly for cattle (Orthoefer et al., 2003). Maize has also become a key player in the agricultural economies of many countries, replacing crops like sorghum and millet (Ayeni et al., 2012).

In Pakistan, maize ranks as the fourth most important cash crop (Olaniyan, 2015). It is cultivated on 1,318,000 hectares, yielding a total of 6,309 metric tons annually. Maize is increasingly utilized by the wet milling and feed industries in Pakistan and is grown extensively across the country’s major agricultural areas. Punjab and Khyber Pakhtunkhwa (KPK) are the leading maize-producing provinces, with spring and autumn being the optimal cultivation seasons. The low output is primarily due to the lack of supportive activities and services, such as artificial insemination methods, along with inadequate and unbalanced nutrition, poor reproductive capacity, and the emergence of various diseases (Lamichhane et al., 2019).

Maize is also a significant dietary source of cadmium (Cd) for humans (Hanif and Akhtar, 2016). It is commonly used by arbuscular mycorrhizal fungi (AMF) as an efficient colonizer and in the phyto-management of polluted soils, particularly those contaminated with Cd (Anjum et al., 2016). Despite Cd stress, maize maintains robust biomass production (Cao et al., 2017). Numerous efforts to remediate HM-contaminated soils have yet to fully restore the environment, highlighting the urgent need for straightforward and economical solutions to control pollutants, reduce plant absorption, and enhance crop tolerance.

Maize faces various biotic and abiotic challenges throughout its lifecycle, with HM-induced soil pollution becoming a critical global environmental issue (Rizwan et al., 2017). Cd is among the most hazardous HMs due to its detrimental effects on plant growth and development. Soils contaminated with cadmium often result from excessive use of fertilizers, chemical sprays, composting in urban areas, and irrigation with industrial water. Cd’s oxidizing properties lead to non-redox stress at the cellular level, causing protein oxidation, lipid peroxidation, and ultimately cell death (Yang et al., 2004). While small amounts of cadmium may not harm plants, higher concentrations, such as cadmium chloride, can impede all stages of plant growth, including root development (Gill et al., 2011). Cd intoxication can also cause complete cell death and cell shrinkage, like the yellowing of plant leaves caused by iron deficiency.

HMs are known for their toxicity and pose significant risks even at low concentrations, often associated with contamination and potential eco-toxicity (Jiang et al., 2001). Improper waste management can lead to the contamination of water and soil by heavy metals (HMs) like lead (Pb), manganese (Mn), cadmium (Cd), and nickel (Ni), originating from various sources including industrial, hygienic, and general human activities (Saletnik et al., 2016). These HMs are particularly hazardous due to their non-biodegradable nature, toxicity to certain crops, and high bioavailability (Sonone et al., 2020). Pb, for instance, can impair chloroplast structure, diminishing light capture and photosynthetic capacity. Additionally, lead interferes with the uptake of essential nutrients like calcium, magnesium, and potassium, impacting plant development and growth. Its toxicity induces the generation of reactive oxygen species (ROS), leading to oxidative stress and damage to essential biological components such as proteins, lipids, and DNA (Del Buono et al., 2022). Pb can also modulate gene expression in plants, altering the expression of crucial proteins and enzymes necessary for plant growth and development. By employing bacterial inoculants, such as plant growth-promoting rhizobacteria (PGPR), presents a viable strategy to boost plant growth and enhance drought resistance. These beneficial microbes augment a plant’s resilience to various abiotic stressors like salt, drought, nutrient deficiency, and metal contamination. Through diverse mechanisms, PGPR bolsters plant development and tolerance, evolving to withstand osmotic stress via direct and indirect pathways. Consequently, utilizing bacterial inoculants emerges as a pragmatic and economically sustainable approach to bolster food security amidst drought conditions (Xu et al., 2012). This study focuses on elucidating the intricate mechanisms through which drought impacts plant growth and how plants adapt to water scarcity, with a specific emphasis on harnessing PGPR in drought-prone regions to enhance crop productivity, growth, and tolerance. The integration of effective microorganisms into bacterial fertilizers represents an appealing and ecologically sound strategy to enhance plant growth and vitality across various environmental contexts (Kour and Yadav, 2022).

Biochar (BC) presents a promising strategy to address Pb, Cd toxicity and drought stress (DS). Its increased porosity and surface area make it effective in enhancing soil water retention capacity (Ćwieląg-Piasecka et al., 2023). This property is particularly beneficial in drought-prone areas where maintaining consistent soil moisture levels is crucial for supporting plant growth under water-deficient conditions (Weifeng et al., 2019). Additionally, BC’s surface characteristics aid in immobilizing Pb and Cd ions in the soil matrix through adsorption mechanisms, thereby reducing their mobility and uptake by plants (Junjie et al., 2020). The dual capability of BC to mitigate DS and alleviate Pb, Cd toxicity highlights its potential as a sustainable soil amendment (Lukáš et al., 2016; Bilal et al., 2022).

The increasing contamination of agricultural soils with HMs poses a significant threat to global food security and environmental sustainability. HMs such as Cd and Pb are persistent pollutants that accumulate in soils, adversely affecting plant growth and development. Recently, there has been growing interest in exploring sustainable and eco-friendly approaches to mitigate the detrimental effects of HMs stress on crops. Among these approaches, BC has emerged as a promising soil amendment due to its unique physicochemical properties and its ability to immobilize HMs, thereby reducing their bioavailability to plants. BC, a carbon-rich material produced from the pyrolysis of organic biomass, has been shown to enhance soil fertility, water retention, and nutrient cycling while mitigating the toxic effects of HMs on plant growth. Despite the increasing body of research on BC’s potential in HMs stress mitigation, there remains a need for comprehensive studies to elucidate its mechanisms of action and optimize its application strategies in agricultural syshoots (Shivvendra et al., 2023).

Therefore, this study aims to investigate the efficacy of BC in combination with GA3 as a novel intervention for enhancing maize tolerance to Cd, Pb and DS, focusing on key growth parameters such as germination percentage, root length, and shoot length. By elucidating the role of BC in alleviating HM stress and improving crop productivity, this research seeks to contribute to the development of sustainable agricultural practices that promote food security and environmental resilience. This study hypothesizes that the incorporation of BC, in conjunction with GA3, can enhance maize tolerance to cadmium stress, thereby improving key growth parameters. The research objectives are twofold: first, to investigate the efficacy of the BC-GA3 intervention in enhancing maize tolerance to Cd, Pb stress, focusing on germination percentage, root length, and shoot length as key growth parameters; second, to elucidate the mechanisms underlying the beneficial effects of BC in alleviating HMs stress and improving crop productivity. To achieve these objectives, the study employs a comprehensive experimental approach, including controlled environment studies and physiological assessments, to evaluate the impact of BC-GA3 intervention on maize growth under Cd, Pb stress. By advancing our understanding of BC’s role in HM stress mitigation and crop enhancement, this research aims to inform evidence-based agricultural practices that promote food security and environmental sustainability.

## 2 Materials and methods

### 2.1 Experimental layout and location

An experiment was conducted in Botany Department, The Islamia University of Bahawalpur to investigate the synergistic approaches aimed at enhancing maize tolerance to Cd, Pb toxicity and DS through the interaction of BC, GA3 and RB. The experimental design employed a complete randomized design (CRD) with three replications. Eighty-four pots, each measuring approximately 18 cm in height and 22 cm in diameter, were filled with soil. Before sowing, seeds were sorted and only healthy seeds were selected for planting.

### 2.2 Soil sampling and analysis

The soil samples were characterized by sandy loam soil with calcareous properties, renowned for its fertility and low organic matter content. Following collection, these samples underwent the identification of trace metal ions (Hashem et al., 2016). Initially, 1g of dried soil was placed in an Erlenmeyer flask, after which 10 ml of HNO_3_ was added, allowing the mixture to stand overnight. Subsequently, controlled heating to 200°C was applied to the flask, followed by cooling and treatment with a mixture of HNO_3_ and HClO_4_. Further heating at 280°C was continued until fumes from HClO_4_ were observed. After cooling, HCl was added, followed by another heating-cooling cycle. The resulting solution was filtered using Whatman filter paper number 42 after being mixed with 1% HCl.

### 2.3 Biochar composition

To produce biochar (BC), leftover fruit and vegetable debris were collected from a nearby market located at 30°11’29.8”N, 71°28’48.8”E. The collected debris was cut into small pieces and subjected to sun-drying. Subsequently, the debris underwent pyrolysis at a temperature of 325 ± 5°C in an aerobic environment. The resulting BC was washed with tap water to remove impurities. To ensure the complete removal of residual ash, the BC was thoroughly rinsed with deionized water. Following this, the BC was dried completely in a well-ventilated area. Once dried, the BC was properly stored for future use. The characteristics of the biochar produced are detailed in **Table 1**.

**Table 1:** Pre-experimental features of irrigation, biochar, and soil.

Soil	Values	Biochar	Values	Irrigation	Values
pH	8.19	pH	8.17	pH	6.71
EC*e* (dS/m)	3.21	EC*e* (dS/m)	3.21	EC (µS/cm)	435
SOC (%)	0.45	Volatile Matter (%)	29	Carbonates (meq./L)	0.00
TN (%)	0.03	Fixed carbon (%)	41	Bicarbonates (meq./L)	4.46
EP (mg/kg)	12.89	TN (%)	0.05	Chloride (meq./L)	0.20
AK (mg/kg)	109	TP (%)	0.11	Ca+Mg (meq./L)	2.33
Sand (%)	25	TK (%)	0.21	Sodium (mg/L)	103
Silt (%)	40	TPb (µg/g)	0.01	Texture	Clay Loam
Clay (%)	35	Particle Size	<2 mm		

TN, Total Nitrogen; EP, Extractable Phosphorus; AK, Available Potassium; TPb, Total Lead; CEC, Cation Exchange Capacity; EC, Electrical Conductivity; SOC, Soil Organic Carbon.

### 2.4 Procurement and sterilization of seeds

FH-1036 hybrid seeds were obtained from a local market. Seed sterilization was carried out through a process involving three rinses with 95% ethanol followed by the application of 5% sodium hypochlorite (NaOCl). This procedure entailed immersing the seeds in a NaOCl solution for 30 minutes, followed by three subsequent washes using 95% ethanol.

### 2.5 Treatments

T1: Control (no Cd stress), T2: mild Cd stress (6 mg Cd/kg soil), T3: severe Cd stress (12 mg Cd/kg soil), T4: 10 ppm GA3 (no Cd stress), T5: 10 ppm GA3 + mild Cd stress, T6: 10 ppm GA3 + severe Cd stress, T7: 0.75% BC (no Cd stress), T8: 0.75% BC + mild Cd stress, T9: 0.75% BC + severe Cd stress, T10: 10 ppm GA3 + 0.75% BC (no Cd stress); T11: 10 ppm GA3 + 0.75% BC + mild Cd stress T12: 10 ppm GA3 + 0.75% BC + Severe Cd stress, T13: Control (no DS = 65% field capacity), T14: 0.5% BC (no DS), T15: RB inoculation (no DS), T16: BC+RB (no DS), T17: Control (DS 40% field capacity), T18: 0.5% BC (DS 40% field capacity), T19: RB inoculation (DS), T20: BC+RB (DS), T21: Control (No Pb Stress), T22: 0.5% BC, T23: RB inoculation (no Pb stress), T24: BC+RB, T25: Control (400 Pb mg/kg soil), T26: 0.5% BC, T27: RB inoculation (400 Pb mg/kg soil), T28: BC+RB.

### 2.6 Irrigation and harvesting

The pots were watered using a water gardening shower to ensure optimal growth conditions for the maize crops. Irrigation was carried out at regular intervals throughout the plant’s growth stages, typically involving four to five cycles until the maize reached physiological maturity. Harvesting was conducted according to the V10 Zadoks growth scale. Samples of both shoot and root tissues were collected for data analysis, including measurements of root and shoot length, as well as fresh and dry masses. After collection, shoot and root samples were dried in an oven set to 65°C for 72 hours to determine dry weights. The timing of maize harvesting varied, typically occurring between 70 to 120 days, depending on environmental factors and maize variety.

### 2.7 Parameters

The study measured several parameters to assess plant growth and physiological responses. These included germination percentage, shoot length (cm), root length (cm), shoot protein content (µg g^-1^), shoot phenolics content (µg g^-1^), root protein content (µg g^-1^), root phenolics content (µg g^-1^), total chlorophyll content (mg g^-1^), chlorophyll a content (mg g^-1^) and chlorophyll b content (mg g^-1^).

### 2.8 Measurement of protein contents

One enzyme activity unit (EU) per mg of protein was used to calculate the protein concentration that results in a 50% photoreduction. Using an extinction coefficient (ϵ) of 2.8 mM cm^-1^, the protein activity was computed and reported as EU mg^-1^ (Hashem et al., 2016).

### 2.9 Measurement of phenolic contents

The Folin-Ciocalteu technique was employed to quantify soluble phenolics (Singleton and Rossi, 1965). After incubation at 45°C for an hour, the absorbance of the samples at 750 nm was measured using a spectrophotometer. The blank consisted of Na_2_CO_3_, the Folin-Ciocalteu reagent, and 500 μl of redistilled water. Total phenolics were calculated using a standard curve generated with gallic acid. The results were expressed in milligrams per dry-weight gram.

### 2.10 Measurement of chlorophyll contents

Fresh leaves were homogenized in 80% acetone (v/v). Subsequently, the mixture was filtered and centrifuged to eliminate insoluble particles, and the resulting supernatant was carefully transferred to a fresh test tube. The absorbance of the resulting solution was measured using a spectrophotometer at wavelengths of 663, 645 and 480 nm (Arnon, 1949). Chlorophyll a and chlorophyll b concentrations were calculated using the following formulas:


$$\text{Chlorophyll a }\left(\frac{\text{mg}}{\text{g}}\right)=\frac{\left(12.7\;\times \text{ A}663\right)–\;\left(2.69\;\times \text{ A}645\right)\times \text{V}}{1000\;\times \text{W}}$$

$$\text{Chlorophyll b }\left(\frac{\text{mg}}{\text{g}}\right)=\frac{\left(22.9\;\times \text{ A}645\right)–\;\left(4.68\;\times \text{ A}645\right)\times \text{V}}{1000\;\times \text{W}}$$

where A663 and A645 represent the absorbance values at 663 nm and 645 nm, respectively, V is the volume of the extract (mL), and W is the weight of the sample (g).


Total Chlorophyll (mg/g) = Chlorophyll a + Chlorophyll b

### 2.11 Data analysis

The collected data underwent analysis of variance (ANOVA) to determine the significance of differences between treatment groups, followed by the application of Fisher’s LSD test using OriginPro 2021 software.

## 3 Results

### 3.1 Effects on maize germination and growth under stress

In the absence of Cd stress (0 Cd), the germination rate reached 67%. Application of 10 ppm GA3 led to a 72% increase in germination, while 0.75% BC resulted in a 76% increase. The combined application of GA3 and BC exhibited the most significant enhancement in germination, reaching 86%. Under Cd stress (6 ppm Cd), 10 ppm GA3 induced a 54% increase in germination, while 0.75% BC treatment led to a 57% rise. The combination of GA3 and BC showed a substantial increase in germination, up by 63% compared to the control. At Cd stress (12 ppm Cd), 10 ppm GA3 and 0.75% BC resulted in 46% and 48% increases in germination, respectively. The combined treatment of GA3 and BC demonstrated the highest increase in germination, reaching 51%.

In the absence of Cd stress (0 Cd), the shoot length measured 30.37 cm. Application of 10 ppm GA3 and 0.75% BC increased shoot length by 31.75 cm and 31.43 cm, respectively. The combined application of GA3 and BC produced the most significant improvement in shoot length, reaching 32.34 cm. Under Cd stress (6 ppm Cd), 10 ppm GA3 and 0.75% BC led to shoot length increases of 26.17 cm and 26.96 cm, respectively. The combined treatment resulted in the highest increase in shoot length, measuring 29.86 cm compared to the control. At Cd stress (12 ppm Cd), 10 ppm GA3 and 0.75% BC increased shoot length by 21.52 cm and 22.26 cm, respectively. The combined treatment of GA3 and BC exhibited the greatest increase in shoot length, reaching 24.77 cm.

**Figure 1:**
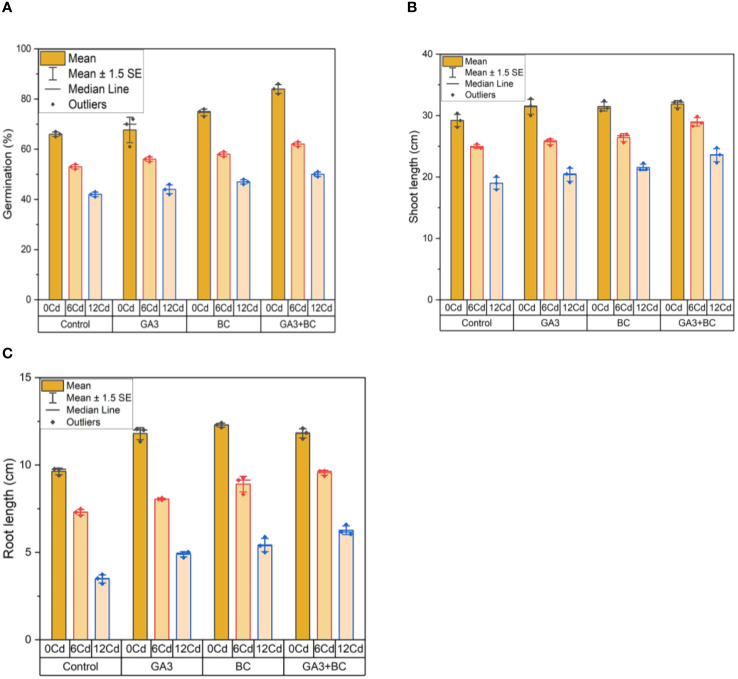
Effect of BC-GA3 intervention in enhancing maize growth parameters under cadmium stress: **(A)** Germination percentage (%) **(B)** Root length (cm) **(C)** Shoot length (cm).

In the absence of Cd stress (0 Cd), the root length was 9.77 cm. Application of 10 ppm GA3 and 0.75% BC resulted in root length increases of 12.00 cm and 12.10 cm, respectively. The combined application of GA3 and BC demonstrated the most significant improvement in root length, reaching 12.41 cm. Under Cd stress (6 ppm Cd), 10 ppm GA3 and 0.75% BC led to root length increases of 8.11 cm and 9.27 cm, respectively. The combined treatment resulted in the highest increase in root length, measuring 19.66 cm compared to the control. At Cd stress (12 ppm Cd), 10 ppm GA3 and 0.75% BC increased root length by 5.02 cm and 5.88 cm, respectively. The combined treatment of GA3 and BC exhibited the greatest increase in root length, reaching 6.59 cm (**Figure 1**).

Under no DS, the application of 0.5% BC led to a substantial 77% increase in germination, while RB resulted in a 64% increase. Notably, the combined administration of BC and RB produced the highest increase in germination at 83%. However, under DS, the germination rates decreased slightly, with 0.5% BC resulting in a 50% increase and RB leading to a 49% increase. Nonetheless, the combination of RB and BC still exhibited the largest increase in germination at 54%.

When subjected to different Pb concentrations, the application of 0.5% BC and RB consistently enhanced germination rates. For instance, at 0 Pb, germination increased by 74% with 0.5% BC and 70% with RB. Remarkably, the combined treatment resulted in the highest germination increase at 87%. Similarly, at 400 ppm Pb, the combined application of BC and RB led to the most substantial increase in germination at 57%.

**Figure 2:**
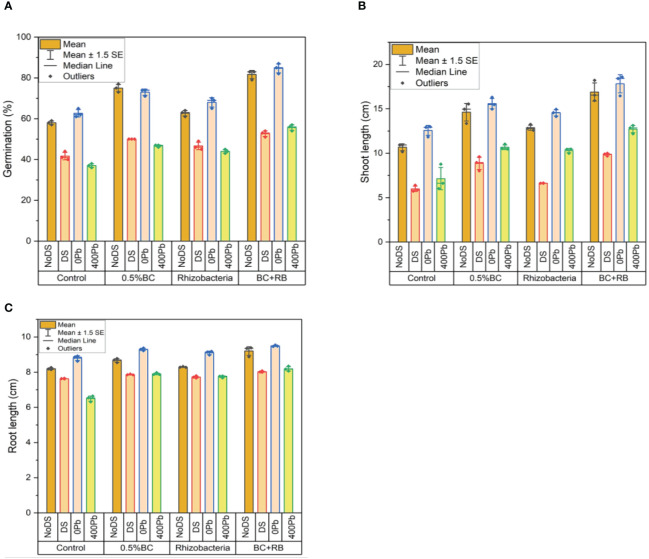
Effect of BC-RB intervention in enhancing maize growth parameters under DS: **(A)** Germination percentage (%) **(B)** Root length (cm) **(C)** Shoot length (cm).

Regarding shoot length, under no DS, 0.5% BC produced a 15.54 cm increase, while RB resulted in a 13.24 cm increase. Once again, the combined application exhibited the greatest enhancement, with a shoot length increase of 18.21 cm. Under DS, although the increases were lower, the combined treatment still outperformed individual applications, resulting in a 9.99 cm increase. In terms of root length, similar trends were observed. Under no DS, 0.5% BC and RB increased root length by 8.77 cm and 8.32 cm, respectively. However, the combined treatment showed the highest increase at 9.38 cm. Under DS, the combined application again led to the most significant increase in root length at 8.07 cm (**Figure 2**).

### 3.2 Treatment effects on maize shoot and root protein content under stress

**Figure 3:**
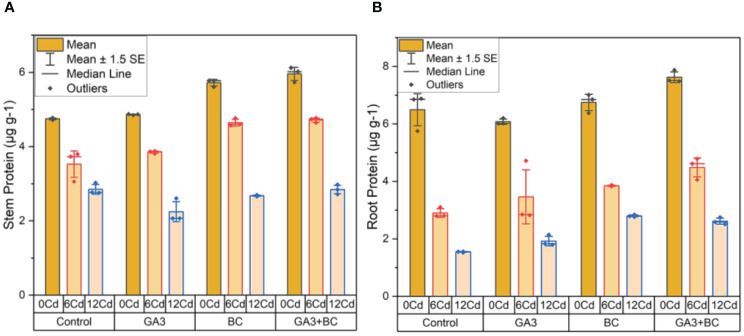
Effect of BC-GA3 intervention in enhancing maize growth parameters under cadmium stress: **(A)** shoot protein µg g^-1^ **(B)** root protein µg g^-1^.

In the absence of Cd stress (0 Cd), the shoot protein content was measured at 4.77µg g^-1^. The application of 10 ppm GA3 resulted in a slight increase to 4.87µg g^-1^, while 0.75% BC led to a more substantial increase to 5.78µg g^-1^. Notably, the combined administration of GA3 and BC exhibited the most significant enhancement in shoot protein content, reaching 6.12µg g^-1^. Under Cd stress (6 ppm Cd), 10 ppm GA3 increased shoot protein content by 3.88µg g^-1^, whereas 0.75% BC resulted in a higher increase of 4.76µg g-1. Interestingly, the combination of GA3 and BC led to a shoot protein content comparable to the control, at 4.77µg g^-1^. At higher Cd stress levels (12 ppm Cd), 10 ppm GA3 and 0.75% BC resulted in increases of 2.60 µg g^-1^ and 2.70µg g^-1^ in shoot protein content, respectively. However, when both GA3 and BC were applied together, the increase in shoot protein content was the most pronounced, reaching 2.98 g/g (**Figure 3A**).

In the absence of Cd stress (0 Cd), the root protein content was recorded at 6.88µg g^-1^. Application of 10 ppm GA3 led to a modest increase of 6.20µg g^-1^ in root protein, while 0.75% BC resulted in a more substantial increase to 7.01µg g^-1^. Notably, the combined application of GA3 and BC exhibited the greatest enhancement in root protein content, reaching 7.87 µg g^-1^. Under Cd stress (6 ppm Cd), 10 ppm GA3 increased root protein content by 4.72µg g-1, while 0.75% BC led to a slightly lower increase of 3.87µg g^-1^. However, when compared to the control, the combined application of GA3 and BC produced the most significant increase in root protein, measuring 4.78µg g^-1^. At higher Cd stress levels (12 ppm Cd), 10 ppm GA3 and 0.75% BC resulted in increases of 2.14µg g-1 and 2.84µg g^-1^ in root protein content, respectively. Yet, the most substantial increase in root protein (2.75 µg g^-1^) was observed when GA3 and BC were administered simultaneously (**Figure 3B**).

**Figure 4:**
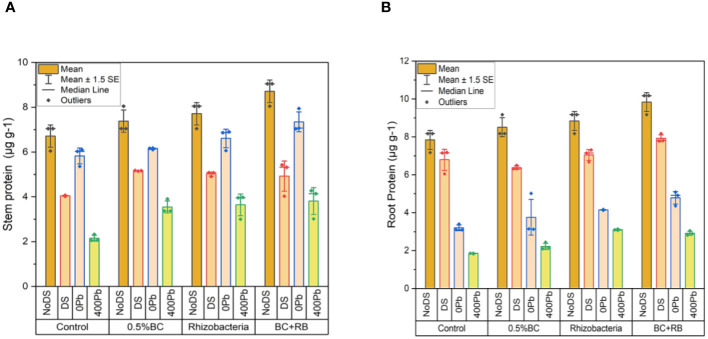
Effect of BC-RB intervention in enhancing maize growth parameters under DS: **(A)** shoot protein µg g^-1^ **(B)** root protein µg g^-1^.

In the absence of DS, the application of 0.5% BC led to an increase of 8.05 µg g^-1^ in shoot proteins, while RB resulted in a 7.05 µg g^-1^ increase. Among all treatments, the combination of RB and BC exhibited the most significant increase in shoot protein content, reaching 9.05 µg g^-1^. Under DS, 0.5% BC resulted in a 5.17 µg g^-1^ increase in shoot protein, whereas RB led to a 5.08 µg g^-1^ increase. Comparatively, the combined application of RB and BC resulted in the highest increase in shoot protein, at 5.32 µg g^-1^ compared to the control. At 0 Pb, 0.5% BC and RB resulted in 6.18 µg g^-1^ and 6.90 µg g^-1^ increases in shoot protein, respectively. The combined application of BC and RB led to the greatest rise in shoot protein (7.94 µg g^-1^). When exposed to 400 ppm Pb, 0.5% BC led to a 3.90 µg g^-1^ increase in shoot protein, while RB resulted in a 3.98 µg g^-1^ increase. However, the combination of BC and RB resulted in the highest increase in shoot protein (4.28 µg g^-1^) compared to the control (**Figure 4A**).

Under conditions of DS, the application of 0.5% BC increased 9.17 µg g^-1^ in shoot proteins, while RB led to an increase of 8.17 µg g^-1^. Among all treatments, the combined application of RB and BC exhibited the most substantial increase in shoot protein content, reaching 10.17 µg g^-1^. Under DS, 0.5% BC resulted in a 6.50 µg g^-1^ increase in shoot protein, whereas RB led to a 7.31 µg g^-1^ increase. The combined treatment of BC and RB resulted in the highest increase in shoot protein (8.17 µg g^-1^) compared to the control. At 0 Pb, 0.5% BC and RB resulted in increases of 5.02 µg g^-1^ and 4.15 µg g^-1^ in shoot protein, respectively. The combined application of BC and RB resulted in a rise in shoot protein (5.80 µg g^-1^). When exposed to 400 ppm Pb, 0.5% BC led to a 2.44 µg g^-1^ increase in shoot protein, while RB resulted in a 3.14 µg g^-1^ increase. The combined treatment of BC and RB produced the largest increase in shoot protein (3.50 µg g^-1^) (**Figure 4B**).

### 3.3 Treatment effects on maize shoot and root phenolics contents under stress

**Figure 5:**
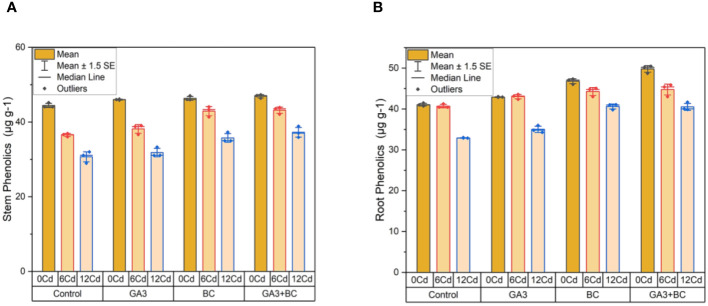
Effect of BC-GA3 intervention in enhancing maize growth parameters under cadmium stress: **(A)** shoot phenolics µg g^-1^ **(B)** root phenolics µg g^-1^.

In the absence of Cd stress (0 Cd), the shoot phenolics content measured 45.18µg g^-1^; the application of 10 ppm GA3 led to a 46.13µg g^-1^ increase, while 0.75% BC resulted in a 47.01µg g^-1^ increase. The most substantial increases in shoot phenolics were observed with the combined treatment of GA3 and BC (47.29 g/g) among all treatments. Under Cd stress (6 ppm Cd), 10 ppm GA3 increased shoot phenolics content by 39.05µg g^-1^, whereas 0.75% BC led to a higher increase of 44.06µg g^-1^. Notably, the combined application of GA3 and BC resulted in the highest increase in shoot phenolics content (43.88µg g^-1^) compared to the control. At higher Cd stress levels (12 ppm Cd), 10 ppm GA3 and 0.75% BC led to increases of 33.30µg g^-1^ and 37.29µg g^-1^ in shoot phenolics, respectively. However, the most significant increase in shoot phenolics (38.88 g/g) was noted when GA3 and BC were applied together (**Figure 5A**).

In the absence of Cd stress (0 Cd), the root phenolics content measured 41.46µg g^-1^; the application of 10 ppm GA3 led to a 42.82µg g^-1^ increase, while 0.75% BC resulted in a 47.34µg g^-1^ increase. The most substantial increases in root phenolics were observed with the combined treatment of GA3 and BC (50.45 g/g) among all treatments. Under Cd stress (6 ppm Cd), 10 ppm GA3 increased root phenolics content by 39.05µg g^-1^, whereas 0.75% BC led to a higher increase of 44.06µg g^-1^. Comparatively, the combined application of GA3 and BC produced the highest rise in root phenolics (43.88µg g^-1^) compared to the control. At higher Cd stress levels (12 ppm Cd), 10 ppm GA3 and 0.75% BC led to increases of 33.30µg g^-1^ and 37.29µg g^-1^ in root phenolics, respectively. However, the most significant increase in root phenolics (38.88 g/g) was observed when GA3 and BC were applied together (**Figure 5B**).

**Figure 6:**
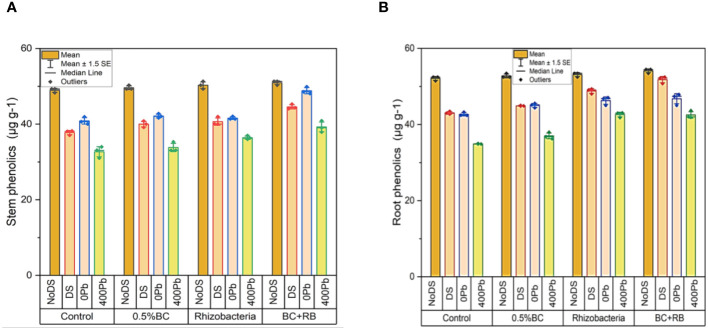
Effect of BC-RB intervention in enhancing maize growth parameters under DS: **(A)** shoot phenolics µg g^-1^ **(B)** root phenolics µg g^-1^.

In the absence of DS 0.5% BC led to a rise of 50.29 µg g-1 in shoot phenolics, while RB caused an increase of 51.09 µg g^-1^. Notably, the combined administration of RB and BC resulted in the highest increase in shoot phenolics, reaching 51.29 µg g^-1^. Under DS, 0.5% BC caused a 40.82 µg g^-1^ increase in shoot phenolics, while RB induced a rise of 41.97 µg g^-1^. Comparatively, the combined treatment of BC and RB led to the most significant increase in shoot phenolics compared to the control, reaching 45.29 µg g^-1^. At 0 Pb, 0.5% BC, and RB increased shoot phenolics by 42.79 µg g^-1^ and 41.36 µg g^-1^, respectively. The application of BC and RB together resulted in the highest elevation in shoot phenolics (49.88 µg g^-1^). In the case of 400 ppm Pb, 0.5% BC and RB induced increases of 35.30 µg g^-1^ and 36.98 µg g^-1^ in shoot phenolics, respectively. Notably, the combination treatment of BC and RB caused the most substantial rise in shoot phenolics (40.88 µg g^-1^) compared to the control (**Figure 6A**).

In the absence of DS, 0.5% BC led to an increase of 53.45 µg g^-1^ in root phenolics, while RB resulted in a rise of 52.45 µg g^-1^. Remarkably, the combined administration of RB and BC produced the greatest elevation in root phenolics, reaching 54.45 µg g^-1^. Under DS, 0.5% BC induced a rise of 45.03 µg g^-1^ in root phenolics, while RB led to an increase of 49.34 µg g^-1^. The treatment of RB and BC together resulted in the highest increase in root phenolics (52.45 µg g^-1^) compared to the control. At 0 Pb, 0.5% BC and RB increased root phenolics by 45.55 µg g-1 and 47.06 µg g^-1^, respectively. When BC and RB were applied together, the most substantial elevation in root phenolics (47.87 µg g^-1^) was observed. In the case of 400 ppm Pb, 0.5% BC and RB induced increases of 37.97 µg g^-1^ and 40.05 µg g^-1^ in root phenolics, respectively. Comparatively, the combined application of BC and RB resulted in the highest increase in root phenolics (43.66 µg g^-1^) (**Figure 6B**).

### 3.4 Treatment effects on chlorophyll a, chlorophyll b and total chlorophyll under stress

**Figure 7:**
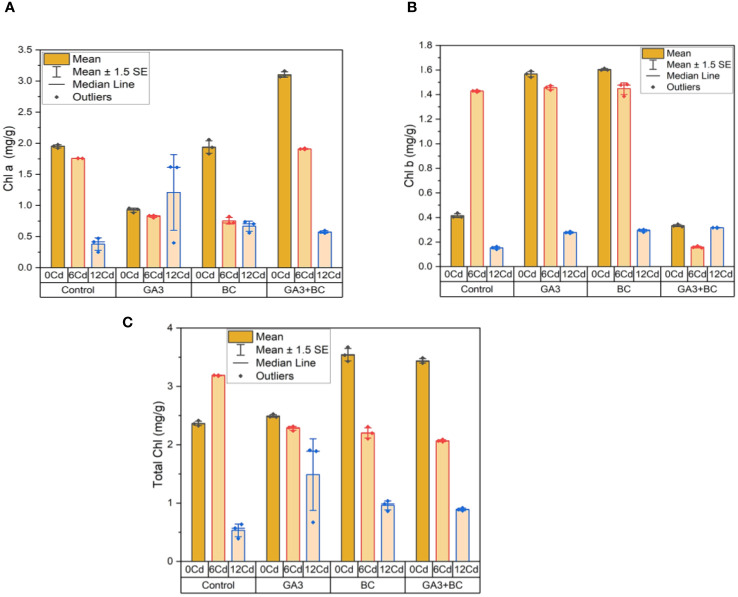
Effect of BC-GA3 intervention in enhancing maize growth parameters under cadmium stress: **(A)** chlorophyll a (mg/g) **(B)** chlorophyll b (mg/g) **(C)** total chlorophyll (mg/g).

In the absence of Cd stress (0 Cd), a chlorophyll a content of 1.98 mg/g was observed. An increase of 0.96 mg/g in chlorophyll a was brought about by the application of 10 ppm GA3, while a more substantial rise of 2.06 mg/g was achieved with 0.75% BC. Notably, the greatest increases in chlorophyll a, reaching 3.16 mg/g, were achieved through the combined administration of GA3 and BC. Conversely, under Cd stress (6 ppm Cd), an increase of 0.84 mg/g in chlorophyll a was induced by 10 ppm GA3, while a rise of 0.82 mg/g was observed with 0.75% BC. The most significant elevation in chlorophyll a, reaching 1.92 mg/g above the control, was observed with the combination treatment of GA3 and BC. At higher Cd stress levels (12 ppm Cd), increases of 0.40 mg/g and 0.74 mg/g in chlorophyll a were observed with 10 ppm GA3 and 0.75% BC, respectively. Remarkably, the application of GA3 and BC together led to the highest increase in chlorophyll a content, reaching 0.60 mg/g (**Figure 7A**).

In the absence of Cd stress (0 Cd), a chlorophyll b content of 0.44 mg/g was recorded. An increase of 0.34 mg/g in chlorophyll b was induced by the application of 10 ppm GA3, while a rise of 0.29 mg/g was achieved with 0.75% BC. Notably, the greatest increases in chlorophyll b, reaching 1.59 mg/g, were observed through the combined administration of GA3 and BC. Under Cd stress (6 ppm Cd), an increase of 1.47 mg/g in chlorophyll b was induced by 10 ppm GA3, while a rise of 0.17 mg/g was observed with 0.75% BC. The highest increase in chlorophyll b, rising by 1.48 mg/g compared to the control, was observed when both GA3 and BC were applied together. At higher Cd stress levels (12 ppm Cd), increases of 0.29 mg/g and 0.30 mg/g in chlorophyll b were observed with 10 ppm GA3 and 0.75% BC, respectively. The most substantial increase in chlorophyll b, reaching 0.32 mg/g, was seen when GA3 and BC were administered together (**Figure 7B**).

In the absence of Cd stress (0 Cd), a total chlorophyll content of 2.41 mg/g was observed. An increase of 2.52 mg/g in total chlorophyll was brought about by the application of 10 ppm GA3, while a rise of 1.61 mg/g was achieved with 0.75% BC. Remarkably, the largest increases in total chlorophyll, reaching 3.49 mg/g, were achieved through the combined administration of GA3 and BC. Under Cd stress (6 ppm Cd), increases of 2.30 mg/g and 2.09 mg/g in total chlorophyll were observed with 10 ppm GA3 and 0.75% BC, respectively. The use of GA3 and BC together resulted in the most substantial rise in total chlorophyll, reaching 2.31 mg/g compared to the control. At higher Cd stress levels (12 ppm Cd), increases of 0.92 mg/g and 1.04 mg/g in total chlorophyll were observed with 10 ppm GA3 and 0.75% BC, respectively. Notably, the combined application of GA3 and BC resulted in the largest increase in total chlorophyll, reaching 1.91 mg/g (**Figure 7C**).

**Figure 8:**
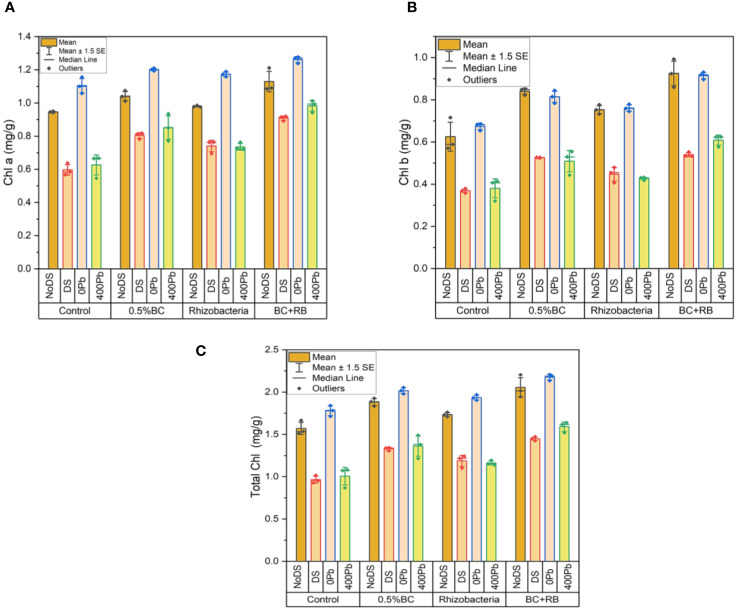
Effect of BC-RB intervention in enhancing maize growth parameters under DS: **(A)** chlorophyll a (mg/g) **(B)** chlorophyll b (mg/g) **(C)** total chlorophyll (mg/g).

In the absence of DS, the application of 0.5% BC led to an increase of 1.0748 mg/g in chlorophyll a (Chl a), while RB induced a rise of 0.9851 mg/g in Chl a content. Notably, the combined treatment of RB and BC resulted in the most substantial increase in Chl a, reaching 1.2114 mg/g. Conversely, under DS, 0.5% BC led to a 0.8176 mg/g increase in Chl a, while RB induced a rise of 0.7636 mg/g. Compared to the control, the simultaneous application of BC and RB caused the largest increase in Chl a, measuring 0.9193 mg/g. At 0Pb, 0.5% BC and RB resulted in increases of 1.2101 mg/g and 1.1896 mg/g in Chl a, respectively. However, the most significant increase in Chl a (1.2771 mg/g) was observed when BC and RB were combined. At 400 ppm Pb, 0.5% BC induced a 0.9351 mg/g increase in Chl a, while RB resulted in a 0.7624 mg/g increase. Remarkably, the combination treatment of BC and RB yielded the highest rise in Chl a (1.0125 mg/g) compared to the control (**Figure 8A**).

In the absence of DS, chl b increased by 0.8512 mg/g with the addition of 0.5% BC and by 0.7764 mg/g with the addition of RB. Notably, the simultaneous application of BC and RB produced the most substantial increase in chl b, rising by 0.9925 mg/g. Conversely, under DS, a rise of 0.5265 mg/g in chl b was observed with 0.5% BC, while RB induced an increase of 0.5636 mg/g. The most significant elevation in chl b, by 0.7526 mg/g over the control, was achieved with the combination treatment of BC and RB. At 0Pb, 0.5% BC and RB resulted in increases of 1.8442 mg/g and 0.7792 mg/g in chl b, respectively. Interestingly, the combination of BC and RB led to the highest increase in chl b (2.2074 mg/g). At 400 ppm Pb, a rise in chl b of 0.5548 mg/g was observed with 0.5% BC, while RB induced an increase of 0.4319 mg/g. Remarkably, the simultaneous application of RB and BC resulted in the highest rise in chl b (0.6251 mg/g) compared to the control (**Figure 8B**).

In the absence of DS, the application of 0.5% BC increased total chlorophyll by 1.926 mg/g, while RB induced a rise of 1.7615 mg/g. Notably, the simultaneous application of RB and BC produced the highest increase in total chlorophyll, reaching 2.2039 mg/g. Under DS, 0.5% BC increased total chlorophyll by 1.3441 mg/g, while the application of RB resulted in a rise of 1.2429 mg/g. Remarkably, the combination treatment of BC and RB resulted in the most substantial increase in total chlorophyll, rising by 1.4719 mg/g compared to the control. At 0 Pb, the increases in total chlorophyll were 2.0543 mg/g and 1.9688 mg/g with 0.5% BC and RB, respectively. However, the highest increase in total chlorophyll (2.2074 mg/g) was observed when BC and RB were applied simultaneously. At 400 ppm Pb, the application of 0.5% BC resulted in an increase of 1.4899 mg/g in total chlorophyll, while RB induced a rise of 1.1943 mg/g. Remarkably, the simultaneous application of RB and BC led to the highest increase in total chlorophyll, rising by 1.6376 mg/g compared to the control (**Figure 8C**).

## 4 Discussion

The research elucidated the intricate interplay among Cd, Pb and DS, BC and GA3 and their impact on shoot protein levels in maize plants. HMs such as lead disrupt the structural integrity of the plasma membrane, leading to alterations in protein fractions and facilitating their infiltration into plant cells (Abedinzadeh et al., 2020). Our investigation revealed that the application of GA3 alone, particularly under non-stressful conditions (0Cd), resulted in a notable increase in shoot protein content. This observation emphasizes the role of GA3 in boosting the plant’s defense mechanisms against Cd stress. These findings are consistent with previous studies (Afrasayab et al., 2010), where the individual or combined administration of Rhizobium and GA3 led to increased protein levels. Additionally, research showed GA3’s capacity to augment protein content in *C. vulgaris* cultures exposed to HMs, thus corroborating our findings (Govarthanan et al., 2016).

Regarding BC treatment, it is evident that the elevation in antioxidant levels induced by BC exerts a crucial influence in impeding the initiation of oxidative chain reactions. Consequently, this mechanism serves to retard or prevent the oxidation of various vital constituents within plant cells, including proteins and lipids (Rafique et al., 2021). Our study’s findings regarding the adverse impacts of Pb and Cd on protein accumulation align with established research, thus offering insights into the underlying mechanisms. Consistent with our observations, previous studies have documented reductions in soluble protein accumulation induced by Pb and Cd exposure in chamomile plants, implying that Cd exposure might stimulate active protein metabolism and provoke potential oxidation processes (Falkowska et al., 2011). The pronounced affinity of Cd for nitrogen and sulfur-containing compounds such as proteins, ligands, and amino acids can result in its binding with these molecules, leading to ion leakage and perturbation of membrane ion channels (Mehdizadeh et al., 2019). These mechanisms collectively contribute to the overall decline in protein content under Cd and Pb stress conditions.

Our investigation revealed an intriguing phenomenon regarding phenolic compounds, manifesting as an elevation in their levels within maize leaves subjected to both Cd and Pb stress. This observation stands in contrast to certain previous studies, which reported an augmentation of total phenolics at lower Cd doses but a reduction at higher concentrations (Kovacik and Backor, 2007). This discrepancy could potentially shoot from species-specific responses or the intricate interplay between Cd exposure and other biochemical pathways inherent to maize physiology. The application of GA3 consistently increased leaf protein levels across all Cd concentrations, counteracting the detrimental effects of Cd on protein content. This positive modulation of protein synthesis by GA3 aligns with previous studies demonstrating enhanced protein levels following RB inoculation and even greater enhancements with GA3 treatment. Similarly, while total soluble protein levels increased notably in treatments involving BC and ascorbic acid, the combination of GA3 and BC consistently outperformed other treatments, highlighting their synergistic potential in augmenting protein content (Dad et al., 2021). Our findings elucidate the intricate interplay between Cd stress, GA3, BC, and protein levels in maize plants, underscoring the potential of BC and GA3, and their combined application, to mitigate the deleterious effects of cadmium stress and promote protein synthesis, thereby offering promising avenues for enhancing crop resilience in challenging environmental conditions and contributing to sustainable agriculture. Furthermore, our investigation into root phenolic content under Cd stress aligns with prior research, emphasizing the significant influence of various treatments on phenolic compounds in plants. The combination treatment of *T. asperellum* and BC demonstrated a notable increase in root phenolics in spring maize, showcasing the versatility of BC in enhancing phenolic production under stress conditions (Kisa et al., 2016). Additionally, integrated approaches involving BC effectively boosted total root phenolic levels, potentially fortifying plant defense mechanisms against HM stress (Yaseen et al., 2021).

The consistent demonstration of BC’s ability to enhance phenolic content underscores its potential as a valuable adjunct for augmenting plant resilience to metal stress [37]. Secondary metabolites such as proline, flavonoids, and phenolics play pivotal roles in mediating plant responses to metal stress by serving as integral components of the plant’s defense mechanisms. These compounds are essential for enabling plants to withstand the deleterious effects of HMs (Amanullah and Khan, 2023). Cultivation of *Z. mays* is confronted with numerous challenges, notably HMs toxicity resulting from Pb contamination and DS. These stressors pose significant threats to plant development and agricultural productivity.

A potential strategy to enhance plant resilience to these stressors involves the application of ameliorative agents such as BC and RB (Dad et al., 2020). The discussion herein investigates the methodologies and outcomes of employing RB and BC to augment *Z. mays*’ resistance to Pb stress and drought. Both Pb stress and drought induce the generation of reactive oxygen species (ROS) within plant cells. ROS, including superoxide radicals, hydrogen peroxide, and hydroxyl radicals, possess high reactivity and can inflict damage on lipids, proteins, and DNA, among other biological constituents (Mumtaz et al., 2021).

The primary effect of Pb stress and drought on plants manifests as a reduction in chlorophyll concentration, crucial for photosynthesis. Diminished chlorophyll content leads to lowered photosynthesis rates and decreased energy production, ultimately impeding plant productivity and growth (Haider et al., 2021). DS and Pb pollution induce chlorophyll damage through oxidative stress, membrane impairment, and ion toxicity. Our findings indicate significant impacts of both DS and Pb stress on electrolyte leakage in maize plants.

DS disrupts cellular water balance, eliciting physiological responses such as stomatal closure, diminished photosynthesis, and the accumulation of osmoprotectants. This imbalance renders cell membranes more permeable, allowing ions and solutes to leak out and cause cellular damage. Conversely, Pb stress triggers maize plants to release more electrolytes than usual, a phenomenon attributed to a process compromising the stability and fluidity of cell membranes. ROS produced by lead and through mechanisms such as lipid peroxidation, further exacerbate cellular damage to membranes and other biological components.

Recent studies have demonstrated the efficacy of PGPR in mitigating the detrimental effects of various abiotic stresses, including HMs and DS. The presence of ACC deaminase, indole-3-acetic acid (IAA), and siderophore in PGPR significantly alleviates the impacts of HMs and drought on plants (Hasanuzzaman et al., 2021). Our investigation revealed a substantial enhancement in plant growth in Pb and DS environments upon the addition of RB. RB likely facilitate this beneficial effect through the production of siderophore, IAA, and ACC deaminase.

ACC deaminase, a prominent enzyme synthesized by PGPRs, plays a crucial role in mitigating the harmful effects of ethylene accumulation, a phenomenon prevalent during drought and HM stress (Naz et al., 2019). Notably, the combined application of stresses, RB, and BC treatments did not yield a statistically significant impact on growth rate, suggesting that each element exerts a stronger individual effect than their combined influences. These findings underscore the critical importance of considering stress factors in agricultural settings, such as DS and Pb, Cd contamination, as they can severely impede plant development and productivity.

RB and BC, as ameliorative treatments, demonstrate substantial impacts, emphasizing their potential to expedite development under stressful conditions. The utilization of beneficial RB facilitates plant development by augmenting nutrient absorption, hormone synthesis, and syshootic resistance. Conversely, BC enhances soil structure, water-holding capacity, and nutrient retention, thereby fostering plant growth. RB contributes to the mitigation of ethylene buildup by producing ACC deaminase, thereby maintaining root growth and delaying senescence, consequently enhancing plant growth. Additionally, RB produces IAA, a plant hormone that stimulates root growth and development, in conjunction with ACC deaminase. Under DS and HMs stress, plant growth is typically hindered due to diminished root growth and development. By synthesizing IAA, RB facilitate root growth and development, thereby ameliorating plant growth and stress tolerance (Bashir et al., 2020). Furthermore, siderophore, another metabolite produced by RB, may contribute to alleviating the detrimental effects of DS and HMs stress on plants. Siderophore acts as an iron-chelating molecule, enhancing the availability of iron to plants (Danish and Zafar-ul-Hye, 2019).

This research advances our understanding of the synergistic effects of Cd, Pb, DS, BC, and GA3 on protein and phenolic content in maize plants, revealing novel insights into their combined potential to mitigate HM stress. By demonstrating that GA3 alone or in conjunction with BC can significantly enhance protein levels and increase phenolic content, our findings highlight the efficacy of these treatments in bolstering plant resilience. This study not only underscores the importance of these agents in enhancing plant defense mechanisms but also provides a foundational basis for developing integrated strategies to improve crop tolerance to environmental stressors, thereby contributing valuable knowledge to the fields of plant physiology and sustainable agriculture. Future research should focus on long-term field trials to evaluate the effectiveness of these treatments in real-world agricultural settings, exploring the mechanisms underlying their synergistic effects, and assessing their impact on other crops and environmental conditions to broaden their applicability and ensure sustainable agricultural practices.

The results of this study demonstrate the significant impact of treatment combinations involving BC, GA3 and RB on maize germination, growth and biochemical responses under Cd, Pb and DS. In the absence of Cd stress, the combined application of GA3 and BC resulted in remarkable enhancements across various parameters, including germination rates, shoot and root length, protein and phenolics content, as well as chlorophyll levels. Similarly, under DS conditions, the synergistic effects of BC and RB led to substantial improvements in maize growth and physiological processes. These findings emphasize the efficacy of integrated approaches in agricultural practices to mitigate the adverse impacts of environmental stressors and enhance crop resilience and productivity. Implementation of such multifaceted strategies holds promise for sustainable agriculture by optimizing crop performance and alleviating the detrimental effects of stress conditions. Further research is recommended to elucidate the underlying mechanisms and refine application protocols for maximizing the benefits of these interventions in real-world agricultural settings.

## Data availability statement

The original contributions presented in the study are included in the article/supplementary material. Further inquiries can be directed to the corresponding authors.

## Author contributions

TA: Conceptualization, Data curation, Formal analysis, Writing – original draft. HQ: Conceptualization, Writing – review & editing. MSA: Investigation, Methodology, Project administration, Writing – original draft. EHS: Investigation, Methodology, Project administration, Writing – review & editing. HF: Software, Supervision, Validation, Visualization, Writing – original draft. WZ: Validation, Visualization, Writing – review & editing. BAA: Methodology, Project administration, Resources, Software, Writing – original draft. MFS: Software, Supervision, Validation, Visualization, Writing – original draft.
